# Paravertebral and Retroperitoneal Vascular Tumour Presenting with Kasabach-Merritt Phenomenon in Childhood, Diagnosed with Magnetic Resonance Imaging

**DOI:** 10.1155/2015/537530

**Published:** 2015-09-08

**Authors:** Gonca Keskindemirci, Deniz Tuğcu, Gönül Aydoğan, Arzu Akçay, Nuray Aktay Ayaz, Ali Er, Ensar Yekeler, Bilge Bilgiç

**Affiliations:** ^1^Department of Pediatric Hematology-Oncology, İstanbul Kanuni Sultan Süleyman Educational and Research Hospital, 34303 Istanbul, Turkey; ^2^Department of Pediatric Hematology-Oncology, Faculty of Medicine, Acıbadem University, 34742 Istanbul, Turkey; ^3^Department of Pediatric Rheumatology, İstanbul Kanuni Sultan Süleyman Educational and Research Hospital, 34303 Istanbul, Turkey; ^4^Department of Radiology, İstanbul Kanuni Sultan Süleyman Educational and Research Hospital, 34303 Istanbul, Turkey; ^5^Department of Radiology, İstanbul Faculty of Medicine, İstanbul University, 34093 Istanbul, Turkey; ^6^Department of Pathology, İstanbul Faculty of Medicine, İstanbul University, 34093 Istanbul, Turkey

## Abstract

Kasabach-Merritt phenomenon (KMP) is characterized by vascular tumour and consumptive coagulopathy with life-threatening thrombocytopenia, prolonged prothrombin time and partial thromboplastin time, hypofibrinogenemia, and the presence of high fibrin split products. We report a case of 3-year-old boy with local aggressive vascular lesions associated with KMP. Magnetic resonance imaging revealed an extensive lesion at paravertebral and retroperitoneal regions that was infiltrating vertebrae. Although we did not get any response to steroid or propranolol treatment, partial response was observed radiologically with interferon-alpha treatment. Unfortunately, the patient died because of the uncontrolled consumptive coagulopathy that led to intracranial hemorrhage which was caused by huge knee hematoma after minor trauma.

## 1. Introduction

Vascular anomalies include a spectrum of disorders from simple lesions to life-threatening entities [1]. Kasabach-Merritt phenomenon is characterized by vascular tumour and consumptive coagulopathy with life-threatening thrombocytopenia, prolonged prothrombin time (PT), and activated partial thromboplastin time (aPTT), hypofibrinogenemia, and the presence of D-dimer and fibrin split products [2]. Kaposiform hemangioendothelioma (KHE) is the responsible lesion most of the time. Mortality rate is around 10%–37% [3]. Here, we describe a 3-year-old boy with unusual localization of extensive vascular lesions involving spinal vertebrae from T11 to L5 that caused fatal consumptive coagulopathy.

## 2. Case

**Figure 1:**
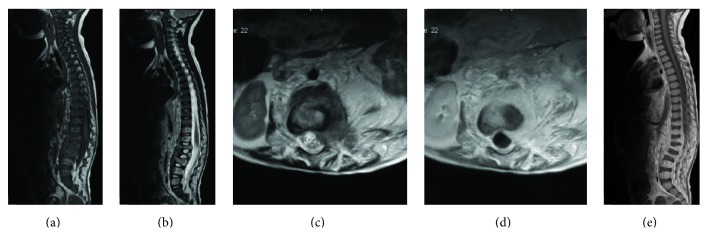
Lesion in the retroperitoneal and paravertebral area and infiltrating vertebrae from T11 to L5. Sagittal MR images show intermediate signal intensity on T1-weighted image (a) and high signal intensity on T2-weighted image (b). Transverse MR image shows high signal intensity on T2-weighted image (c). Transverse and sagittal contrast-enhanced T1-weighted images show homogeneous enhancement of the lesions (d-e).

**Figure 2:**
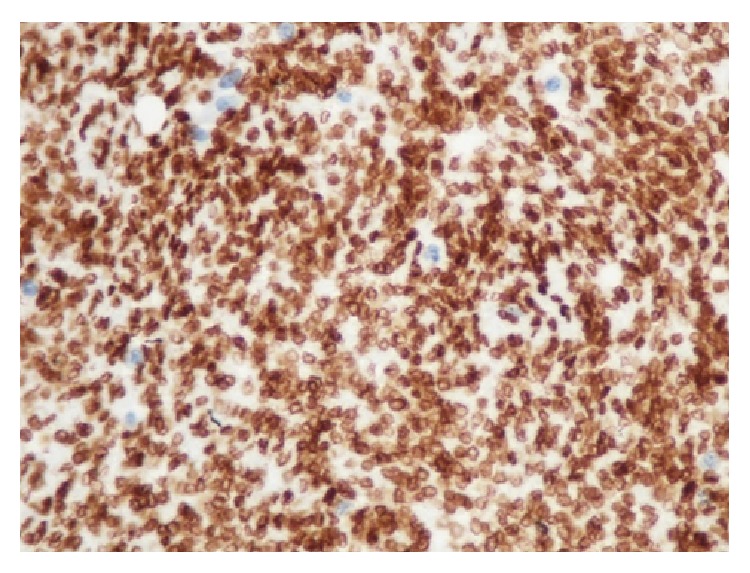
Extensive Glut-1 nuclear positivity in immunohistochemistry staining in biopsy (400x magnification in microscope).

**Figure 3:**
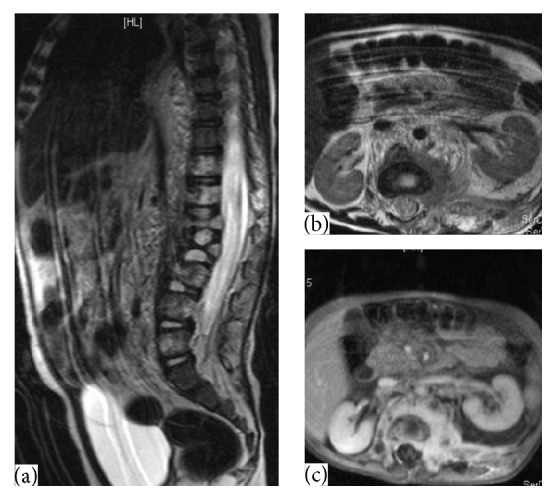
Partial response after interferon-*α* therapy. Sagittal and transverse MR images show high signal intensity on T2-weighted image (a-b). Transverse contrast-enhanced T1-weighted image shows homogeneous enhancement of the lesions (c).

A 3-year-old boy was referred to our hematology clinic because of ecchymosis on the legs and arms for 2 weeks in 2012, July. Medical history was unremarkable. Physical examination revealed ecchymosis on the legs, arms, and back. Muscle atrophy was evident on the upper legs and gluteal region, but there were no vascular lesions on the skin. It was also noted that the child had waddling gait. On the blood count, hemoglobin was 9 gr/dL, leukocyte count was 6100/*μ*L, and platelet count was 7000/*μ*L. On bone marrow examination, megakaryocytes were seen and exclusion of malignant diseases was made. On coagulation study, PT was 38.2 sec, aPTT was 52.4 sec, INR was 4, D-dimer was 2001 mg/dL, and fibrinogen level was 37.7 mg/dL. Abdominal ultrasonography and chest X-ray were normal. Because of the muscle atrophy in the legs and gait disturbance, spinal magnetic resonance imaging (MRI) was taken to exclude neurological problems. Diffusely infiltrating lesion in the paravertebral and retroperitoneal area from T11 to L5 vertebrae with extension into the left side of spinal canal was shown on the lumbar MRI examination. It was variable hypointermediate signal intensity on T1W images and predominantly hyperintense on the T2W images with heterogeneous areas. Postcontrast MRI showed diffuse contrast enhancement of all areas of lesions. The bony structure showed compression on especially central area of L1, L2, and L3 vertebrae corpus, and there was no signal change of the bone marrow of these vertebrae (Figure 1). There was no evidence of any other lesion in the brain or abdomen. With the thrombocytopenia, consumptive coagulopathy, ecchymosis in physical examination, and locally aggressive lesions in the radiologic evaluation, lesion was considered to be vascular lesions, KHE mostly. Prednisolone was started at a dose of 2 mg/kg/day. But, with this treatment, severe thrombocytopenia and abnormal coagulation profile persisted. On the third week, propranolol was added to the treatment at a dose of 2 mg/kg/day. After 2 months of these treatments, clinical, radiological, and laboratory improvements were not observed. To rule out hemangioblastoma, biopsy was taken after the replacement therapies with platelet and fibrinogen infusions. But, just after the surgery, bleeding from the surgical site could not be controlled, and massive amounts of thrombocyte, erythrocyte, fibrinogen, and fresh frozen plasma (FFP) transfusions were needed. Ten hours after the surgery, respiratory distress developed and the patient was transferred to intensive care unit. He was extubated on the sixth day and was taken back to the hematology clinic. Immunohistochemistry staining showed Glut-1 positive erythrocytes in pathological examination and any malign transformation was not detected (Figure 2). Propranolol and corticosteroid treatment did not improve thrombocytopenia and consumptive coagulopathy that was lasting for more than two months; interferon-*α* (IFN-*α*) treatment was started at a dose of 3 million U/m^2^/day subcutaneously. Steroid and propranolol treatments were stopped by tapering. On the third month of IFN-*α* treatment, the follow-up MRI showed moderate regression of lesion size, but there was no significant signal change before and after contrast images (Figure 3) and chronic disseminated intravascular coagulopathy symptoms persisted both clinically and in the laboratory tests. On the 4th month of IFN-*α* treatment, the patient had a minor knee trauma and the next day huge hematoma developed around the knee. Despite thrombocyte, fibrinogen, and FFP transfusions, bleeding in the knee and consumptive coagulopathy could not be kept under control. Two days after this incident, intracranial hemorrhage developed that caused the death of the patient, and while thrombocyte count was 5000/*μ*L, fibrinogen level was 70 mg/dL and PT was 40 sec.

## 3. Discussion

Kasabach-Merritt phenomenon (KMP) is characterized by consumptive coagulopathy and vascular tumour that can be found in the skin, retroperitoneum, mediastinum, pelvis, visceral organs, and mesentery [2]. With the unexplained thrombocytopenia and consumptive coagulopathy, KMP should be considered. The diagnosis of visceral lesions can be difficult, especially when there are no vascular lesions on the skin. Retroperitoneal lesions are often large, easy to be missed clinically, and generally associated with a higher mortality [4, 5]. In the absence of the typical cutaneous findings, imaging techniques can be used to confirm the diagnosis and to determine the extent of the involvement. MRI is the gold standard technique according to the studies in this field [6, 7]. In our case, the diagnosis was made by radiological findings in MRI and we also used the diagnostic criteria for the consumptive coagulopathy developed by ISTH [8].

It has been recently shown that KMP is complicated with variety of vascular tumours not only with infantile hemangioma but also with KHE and tufted angioma [9]. According to the ISSVA classification, vascular lesions associated with thrombocytopenia and consumptive coagulopathy are KHE mostly [1]. On the light of this knowledge, locally aggressive vascular lesion is considered to be KHE in our case. Although Glut-1 is a unique immunohistochemical marker in endothelial cells in infantile hemangiomas, there is a literature of Glut-1 positive in KHE [1, 10]. In KMP treatment, there are two aims. One is the regression of the lesion and the other is controlling and preventing bleeding from thrombocytopenia and coagulopathy [2, 3]. Several treatment approaches are employed; however, it is unclear which is superior and there are no consensus guidelines for the treatment of KMP. Prednisolone is the first line therapy. Most patients respond to the therapy with a dose of 2-3 mg/kg/day within a few days [4]. Higher doses (5 mg/kg/day) or megadoses (30 mg/kg/day, for 3 days) can also be used [4, 11, 12]. The other option is propranolol treatment with a dose of 2 mg/kg/day. The most important advantages of propranolol over corticosteroids are efficacy, safety, fewer side effects, and low cost. In unresponsive cases, IFN-*α* is used to treat rapidly growing, life-threatening hemangiomas at a dose of 3 million U/m^2^ subcutaneously per day. According to the literature, the success of the IFN-*α* treatment is 80% and 90% [13]. However, there are some side effects of IFN-*α* such, as influenza-like symptoms and fever, somnolence, anorexia, diarrhea or constipation, neutropenia, high level of aminotransferases, and neurotoxicity [8, 13, 14]. Because of this, compared to the corticosteroids and propranolol, IFN-*α* should be used for proliferative hemangiomas as a second line therapy. There is currently no consensus on the second line management of KHE that was resistant to prednisone, propranolol, and IFN-*α* [15]. If there is no response to these therapies, cyclophosphamide, vincristine, pentoxifylline, ticlopidine, platelet derived growth factor, and imiquimod can be used [2, 4, 16]. Radiotherapy is an option for life-threatening hemangiomas, but, as it can cause secondary tumors, its use should be evaluated for its safety and efficacy [17, 18]. Surgical excision of hemangiomas is suggested only for cases involving eyelid or huge scalp hemangiomas [16]. Platelet transfusions should only be used when bleeding cannot be controlled or before surgical procedures. Everolimus or sirolimus, an inhibitor of the mammalian target of rapamycin (mTOR), was successfully used recently in the treatment of KHE [15]. Prognosis of KMP is related to lesion's location, size, and depth of invasion.

In our case, with corticosteroid and propranolol therapy, we did not get any response in clinical, radiological, and laboratory parameters. With IFN-*α* treatment, partial response was seen radiologically and the patient died under IFN-*α* treatment. Similar to our case, Hatley et al. were treated from a giant vascular tumour at the retroperitoneal lesion with IFN-*α* [19]. To the best of our knowledge, this is the first childhood vascular tumour with extensive involvement of paravertebral and retroperitoneal area and vertebral corpus diagnosed with MRI that responded partially to the IFN-*α* treatment radiologically. We also emphasize the importance of radiologic evaluation at the differential diagnosis of thrombocytopenia and consumptive coagulopathy without any visible etiological lesion in the skin.
